# Alcohol, No Ordinary Commodity: policy implications for Canada

**DOI:** 10.3389/fpubh.2024.1335865

**Published:** 2024-05-22

**Authors:** Jean-François Crépault, Timothy S. Naimi, Jürgen Rehm, Kevin D. Shield, Samantha Wells, Ashley Wettlaufer, Thomas F. Babor

**Affiliations:** ^1^Centre for Addiction and Mental Health (CAMH), Toronto, ON, Canada; ^2^Dalla Lana School of Public Health, University of Toronto, Toronto, ON, Canada; ^3^Canadian Institute for Substance Use Research, Victoria, BC, Canada; ^4^Alcohol Program, National Center for Chronic Disease Prevention and Health Promotion, Centers for Disease Control and Prevention, Atlanta, GA, United States; ^5^Institute of Medical Science, University of Toronto, Toronto, ON, Canada; ^6^Department of Psychiatry, University of Toronto, Toronto, ON, Canada; ^7^Institute of Clinical Psychology and Psychotherapy & Center for Clinical Epidemiology and Longitudinal Studies, Technische Universität Dresden, Dresden, Germany; ^8^Center for Interdisciplinary Addiction Research (ZIS), Department of Psychiatry and Psychotherapy, University Medical Center Hamburg-Eppendorf (UKE), Hamburg, Germany; ^9^Department of Epidemiology and Biostatistics, Schulich School of Medicine and Dentistry, University of Western Ontario, London, ON, Canada; ^10^School of Psychology, Deakin University, Geelong, VIC, Australia; ^11^Department of Public Health Sciences, University of Connecticut School of Medicine, Farmington, CT, United States

**Keywords:** alcohol, alcohol policy, health policy, public health, substance use

## Abstract

Alcohol is a favorite psychoactive substance of Canadians. It is also a leading risk factor for death and disability, playing a causal role in a broad spectrum of health and social issues. *Alcohol: No Ordinary Commodity* is a collaborative, integrative review of the scientific literature. This paper describes the epidemiology of alcohol use and current state of alcohol policy in Canada, best practices in policy identified by the third edition of *Alcohol: No Ordinary Commodity*, and the implications for the development of effective alcohol policy in Canada. Best practices – strongly supported by the evidence, highly effective in reducing harm, and relatively low-cost to implement – have been identified. Measures that control affordability, limit availability, and restrict marketing would reduce population levels of alcohol consumption and the burden of disease attributable to it.

## Introduction

Alcohol is a favorite psychoactive substance of Canadians. At the same time, alcohol consumption is a leading risk factor for death and disability, playing a causal role in a broad spectrum of health and social issues. Since Confederation, Canada has implemented various approaches to alcohol policy, from prohibition to commercialization, with health often an afterthought.

*Alcohol: No Ordinary Commodity* (1) is a collaborative, integrative review of the alcohol literature. Its third edition, released in November 2022, reviews the latest evidence around alcohol use and related problems, policy measures that reduce harm, and the role of the alcohol industry in the policy-making process. This paper describes the epidemiology of alcohol use and current state of alcohol policy in Canada, best practices in policy identified by the third edition of *Alcohol: No Ordinary Commodity*, and the implications for the development and implementation of effective alcohol policy in Canada.

## Epidemiology of alcohol use and alcohol-related problems in Canada

In 2018, 76.5% of Canadian adults consumed alcohol, with 21.6% engaged in heavy episodic or “binge” drinking (usually defined in Canada as consuming five or more standard drinks on one occasion for men, or four or more standard drinks on one occasion for women) within the past month (2). Although alcohol use and binge drinking rates have remained relatively stable among adults aged 20 and above since 2008, among those aged 15 to 19 the prevalence of past-year alcohol use decreased from 77.3% in 2008 to 46.3% in 2019, with past-month binge drinking decreasing from 34.9% in 2008 to 13.8% in 2019 (2).

Among Canadians who drink, recorded adult (15 years of age and older) *per capita* alcohol consumption (APC) decreased slightly from 8.7 liters of ethanol (alcohol) in 1990 to 8.1 liters of ethanol per adult in 2021, though with substantial regional variation (3). In comparison to global statistics, Canada’s alcohol use exceeds the global average APC of 5.5 liters in 2019, but, including unrecorded consumption, it is similar to the United States’ APC of 9.6 liters (4). Alcohol consumption at the population level, including both APC and binge drinking, has been strongly associated with negative health outcomes (5).

Alcohol use is estimated to cause 18,000 deaths (accounting for 4.7% of all deaths) and 105,000 hospitalizations in Canada each year, primarily from accidents, injuries and chronic diseases like cancer, liver cirrhosis and heart disease (4, 6). There have been recent increases in alcohol-attributable deaths, especially in fully alcohol-attributable causes of death (7). Data indicate that 16.7% of Canadian adults had a lifetime diagnosis of an alcohol use disorder and 2.2% had a past-year diagnosis of an alcohol use disorder (8).

## Alcohol policy options: what works and what doesn’t

Based on a comprehensive review of the alcohol policy literature, *Alcohol: No Ordinary Commodity* (ANOC) sorts alcohol policy measures into three categories, based on the extent to which evidence demonstrates their effectiveness:

- *Best practices.* These measures are highly effective at reducing alcohol-related harm and relatively low-cost to implement.

o Controlling affordability (e.g., through alcohol taxes and pricing measures).
o Limiting availability (e.g., through limits on hours and place of sale, public monopoly on retail sales, and minimum age for purchase).
o Restricting marketing (e.g., a ban of alcohol advertising, marketing, and promotion).

- *Good practices.* These are second-line interventions: measures that are less effective at reducing harms than the best practices but are considered important alcohol policies.

o Some educational activities (e.g., warning labels on beverage containers, campaigns against impaired driving).
o Impaired driving counter-measures (e.g., lower blood alcohol concentration for younger drivers, random breath testing).
o Modifying the drinking environment (e.g., training staff at locations that sell alcohol for on-premise consumption, enhanced enforcement of laws at such locations).
o Treatment and early intervention (e.g., brief counseling interventions, psychosocial treatment, pharmacotherapies).

- *Ineffective/potentially harmful practices.* These are measures ostensibly intended to reduce alcohol-related harms but unlikely to do so. Examples include:

o Industry self-regulation of marketing.
o Industry “responsible drinking” programs.

## The current state of alcohol policy in Canada

In Canada, alcohol policy is conducted at both the federal and provincial/territorial government levels. Recently the Canadian Alcohol Policy Evaluation (CAPE) Project assessed federal alcohol policies across 10 policy domains, and the provinces and territories across 11 policy domains (9). CAPE scores represented the extent to which effective policy measures were in place in each jurisdiction. The federal government earned a failing grade for its alcohol policies with an overall 37% score across the 10 domains. The top two weighted federal domains (pricing / taxation and marketing / advertising controls, both ANOC best practices) received failing grades. Of note, the Canadian federal government does not mandate standard drink labeling or any health warning information. In addition, its advertising compliance code relies principally on self-regulation, is badly outdated (it does not cover digital media), is not supported by the requisite expertise or resources to conduct surveillance to assess compliance, and has an unworkable and ineffective complaint system (10).

Although Canadian provinces and territories vary greatly in their approaches to alcohol policy, none has a particularly effective policy environment. CAPE scores ranged from 32 to 44%, with a mean score of 37% (9). In the three provincial/territorial domains that correspond to ANOC best practices – controlling affordability, limiting availability, and restricting marketing – the provinces and territories fared poorly. In fact, there were only five passing grades (50% or higher) for any of the best practice areas in any province or territory: Prince Edward Island for affordability, Nunavut for availability, and Manitoba, Nunavut, and Québec for marketing.

### Implemented best practices

While all provinces and territories individually fare poorly in terms of effective alcohol policy overall, some effective practices are in place. Some examples of best practices follow.

In terms of controlling affordability, about half of provinces and territories have alcohol-specific taxes at the retail level, meaning that alcohol is taxed more than most other consumer goods in those jurisdictions. In addition, most provinces and territories have some type of minimum pricing scheme for off-premise and/or on-premise sales. (“Off-premise” refers to locations selling alcohol for off-premise consumption, while “on-premise” refers to locations selling alcohol for on-premise consumption, e.g., bars and pubs). For example, a 25% alcohol-specific ‘health tax’ in Prince Edward Island (PEI) applies to all off-premise alcohol sales. PEI also has the highest off-premise minimum prices in Canada, which are updated periodically to reflect inflation. Manitoba’s pricing system is unique in having ethanol-based minimum pricing (per liter of ethanol rather than per liter of beverage) for off-premise sales. Often called “volumetric pricing,” this method ensures that a product’s price is roughly proportional to its ethanol content, creating an incentive to purchase less potent products.

In terms of limiting availability, Saskatchewan and Yukon have population-based limits for off-premise outlet density. Newfoundland & Labrador and the Northwest Territories limit hours of sale to 11 h a day. Nunavut is the only jurisdiction in which all off-premise alcohol sales are conducted by a government retail monopoly.

No Canadian jurisdiction has implemented substantive restrictions on digital alcohol advertising, marketing, and promotion. Five jurisdictions place limits on where alcohol ads can be located (e.g., near schools or in media targeted to youth), five prohibit price-based advertising, and four have banned advertising by third parties (e.g., delivery services).

### Alcohol policy in Canada compared with other countries

The region with the most successful alcohol control policy implementation in the past decade was Eastern Europe. An analysis of measures in the Baltic countries and Poland, including taxation increases that reduced alcohol affordability and availability restrictions of more than 20% in purchasing hours, showed that on average these measures reduced APC by 0.8 liters of ethanol *per capita* (11), all-cause mortality reductions of 2.3% per year among males, and a lesser, non-significant reduction among females (12). The biggest single impact on health was found by the increase in excise taxation in Lithuania in 2017, which prevented more than 1,000 all-cause deaths in the following year (13, 14). In addition, this increase in excise taxation decreased socioeconomic mortality disparities (15). Similarly, Russia reduced APC and alcohol-related as well as all-cause mortality with a mix of policy measures including the best practices described above (16, 17). Implemented measures include limits on alcohol production and availability (mid-1980s), introduction of minimum prices (2003), restrictions on marketing (2004), measures against unrecorded alcohol (2006), a ban on internet sales (2007), and tax increases (2010–2012). These changes have been associated with improvements in life expectancy (18).

In general, public health considerations seem to be more evident in recent alcohol control policies in Europe than in North America. Further examples are the consideration of mandatory warning labels in the European Union, the implementation of such warning labels in Ireland, (19) and the implementation of minimum unit pricing in Scotland and Wales (20). This is in contrast to inaction or loosening of policies in Canada. For example, studies have linked the privatization and expansion of alcohol retail with increased hospitalizations in Ontario (21) and mortality in British Columbia (22). These processes appear to have accelerated since the COVID-19 pandemic.

### Stakeholders and agendas

Many actors are involved in alcohol policy in Canada. Various non-governmental organizations (NGOs) are involved, some concerned with alcohol use in general, others with specific problems such as driving under the influence of alcohol, and yet others that touch alcohol as part of a larger problem (e.g., community NGOs concerned with violence, or initiatives to reduce the burden of non-communicable disease). There are also multiple levels of government with responsibility for different aspects of alcohol policy: addressing availability is primarily a provincial/territorial matter, with some municipal involvement; regulating marketing is mainly a federal issue; while affordability (taxation and, in the case of provinces and territories with public retailers, setting prices) falls under both federal and provincial/territorial jurisdiction. At each level, multiple ministries are involved (e.g., provincial ministries of Finance, Health, Agriculture), which often do not communicate with each other, let alone with NGOs. At every step, the alcohol industry generally attempts to influence the policy process, using a variety of strategies including lobbying, undermining science, and mounting legal challenges (1, 23, 24). Consequently, public health advocates need a “health in all policies” approach to alcohol, (25) which addresses not only potential health outcomes, but alcohol’s economic and social impacts. In a globalized world where the alcohol industry is highly concentrated in a few multinational corporations, there also needs to be a globalized approach to public health: tobacco control has shown the importance of a legally binding treaty or convention, (26) and such a legal instrument seems necessary for alcohol as well (27).

## Implications and recommendations

The implementation of alcohol policy best practices – controlling affordability, limiting availability, and restricting marketing – are not technically complex or difficult to implement. The World Health Organization has for years been referring to these measures as “best buys”: the interventions “considered to be the most cost-effective and feasible for implementation” (28, p 3). Despite substantial evidence for these best practices, all Canadian provinces and territories individually fare poorly in terms of alcohol policy. However, some effective practices are in place. In fact, the CAPE Project found that if a province were to adopt all the best policy practices that are currently in place somewhere in Canada, this hypothetical province would achieve a policy score of 80% (9). Opportunities to adopt effective alcohol policy exist, and provinces and territories need to look no further than their neighbors for examples.

The experiences of countries that have overcome the inertia that characterizes the alcohol policy environment in Canada suggest that the following activities could serve as enablers for the implementation of effective alcohol policy (1):

- Develop capacity among civil society organizations for evidence-based policy advocacy.
- Provide key constituencies and the general public with information on the health, social, and economic benefits of alcohol policy best practices and the costs of inaction or ineffective/ harmful practices.
- Mobilize key health advocates to pay greater attention to alcohol.
- Develop partnerships between academia and civil society organizations to facilitate the dissemination of evidence-based alcohol policy.
- Conduct campaigns to promote taxes on alcohol as a way to finance treatment, prevention, and the health care system more broadly.

## Conclusion

The authors of *Alcohol: No Ordinary Commodity* remind us that “the difference between good and bad alcohol policy is not an abstraction, but very often a matter of life and death” (1, p 326). Alcohol consumption is a leading risk factor for death and disability, responsible for a variety of health and social issues in Canada. Alcohol-attributable deaths have recently been on the rise. But the way to tackle these issues is clearer than ever. Best practices – strongly supported by the evidence, highly effective in reducing alcohol-related harm, and relatively low-cost to implement – have been identified. Measures that control affordability, limit availability, and restrict marketing would reduce population levels of alcohol consumption and, in turn, reduce the burden of disease attributable to alcohol. All that is needed is political will.

## Author contributions

J-FC: Conceptualization, Investigation, Writing – original draft. TN: Investigation, Writing – original draft. JR: Conceptualization, Investigation, Writing – original draft. KS: Investigation, Writing – original draft. SW: Conceptualization, Writing – review & editing. AW: Investigation, Writing – review & editing. TB: Conceptualization, Writing – review & editing.
